# Comparative Analysis of Root Na^+^ Relation under Salinity between *Oryza* *sativa* and *Oryza coarctata*

**DOI:** 10.3390/plants11050656

**Published:** 2022-02-28

**Authors:** Tetsuya Ishikawa, Lana Shabala, Meixue Zhou, Gayatri Venkataraman, Min Yu, Gothandapani Sellamuthu, Zhong-Hua Chen, Sergey Shabala

**Affiliations:** 1 Tasmanian Institute of Agriculture, College of Science and Engineering, University of Tasmania, Hobart, TAS 7005, Australia; tetsuya.ishikawa@utas.edu.au (T.I.); l.shabala@utas.edu.au (L.S.); meixue.zhou@utas.edu.au (M.Z.); 2 Plant Molecular Biology Laboratory, M. S. Swaminathan Research Foundation, III Cross Street, Taramani Institutional Area, Chennai 600113, India; gayatri@mssrf.res.in (G.V.); sellamuthu@fld.czu.cz (G.S.); 3 International Research Centre for Environmental Membrane Biology, Foshan University, Foshan 528000, China; yumin0820@hotmail.com; 4 Forest Molecular Entomology Lab, Excellent Team for Mitigation (ETM), Faculty of Forestry and Wood Sciences, Czech University of Life Sciences Prague, 16500 Prague, Czech Republic; 5 School of Science, Hawkesbury Institute for the Environment, Western Sydney University, Penrith, NSW 2751, Australia; z.chen@westernsydney.edu.au

**Keywords:** rice, salinity, halophyte, root, microelectrode ion flux, MIFE, transporters

## Abstract

Na^+^ toxicity is one of the major physiological constraints imposed by salinity on plant performance. At the same time, Na^+^ uptake may be beneficial under some circumstances as an easily accessible inorganic ion that can be used for increasing solute concentrations and maintaining cell turgor. Two rice species, *Oryza sativa* (cultivated rice, salt-sensitive) and *Oryza coarctata* (wild rice, salt-tolerant), demonstrated different strategies in controlling Na^+^ uptake. Glasshouse experiments and gene expression analysis suggested that salt-treated wild rice quickly increased xylem Na^+^ loading for osmotic adjustment but maintained a non-toxic level of stable shoot Na^+^ concentration by increased activity of a high affinity K^+^ transporter HKT1;5 (essential for xylem Na^+^ unloading) and a Na^+^/H^+^ exchanger NHX (for sequestering Na^+^ and K^+^ into root vacuoles). Cultivated rice prevented Na^+^ uptake and transport to the shoot at the beginning of salt treatment but failed to maintain it in the long term. While electrophysiological assays revealed greater net Na^+^ uptake upon salt application in cultivated rice, *O. sativa* plants showed much stronger activation of the root plasma membrane Na^+^/H^+^ Salt Overly Sensitive 1 (SOS1) exchanger. Thus, it appears that wild rice limits passive Na^+^ entry into root cells while cultivated rice relies heavily on SOS1-mediating Na^+^ exclusion, with major penalties imposed by the existence of the “futile cycle” at the plasma membrane.

## 1. Introduction

Sodium toxicity is considered to be a major constraint affecting plant performance caused by salt stress in the long term. As a result of selective breeding, salinity-tolerant rice cultivars accumulate less Na^+^ in the shoot compared with sensitive cultivars [1,2,3,4,5]. A number of previous studies focused on the mechanism of Na^+^ retrieval back from xylem operated by a high-affinity K^+^ transporter OsHKT1;5 that reduces shoot Na^+^ accumulation in rice [5,6,7,8,9]. Once unloaded from the xylem, Na^+^ needs to be extruded into external medium. Root Na^+^ exclusion is known to be operated by a Na^+^/H^+^ exchanger Salt Overly Sensitive 1 (SOS1) at the root epidermis [10,11], and beneficial effects of enhancement of SOS1 operation fuelled by H^+^-ATPase on salt tolerance in rice was demonstrated [12,13]. In addition to Na^+^ exclusion, increasing biosynthesis of organic osmolytes has been also targeted to improve salinity-induced osmotic stress tolerance in rice [14,15,16]. However, despite numerous attempts, achievements in breeding salinity-tolerant rice are still quite modest [17,18].

If one enhances Na^+^ exclusion by SOS1, then plants need to rely on de novo synthesis of organic osmolytes (compatible solutes) for osmotic adjustment, which comes with a considerable energy cost, leading a depletion of the ATP pool [19,20]. Therefore, SOS1-mediated root Na^+^ exclusion activity did not correlate with overall salinity tolerance in barley [21] and rice varieties [22] when assessed by direct Na^+^ flux measurements using electrophysiological techniques. The efficacy of Na^+^ exclusion strategy mediated by SOS1 in rice is further complicated by the presence of the apoplastic pathway of Na^+^ entry, named as bypass flow. Despite anatomical barriers, bypass flow causes a significant amount of passive Na^+^ entry from sites of lateral root emergence, areas of weak Casparian strip barrier formation, and cell walls near the root apices that has long been considered as a major component of high salt sensitivity in rice [7,23,24,25,26]. Due to this passive Na^+^ entry pathway, SOS1 transporters in rice may operate in a “futile cycle”, depleting energy but not achieving a significant reduction in Na^+^ content. Thus, selection of inappropriate traits (i.e., Na^+^ exclusion and de novo synthesis of compatible solutes) can be the reason of the failure to produce salinity tolerant rice over the past decades.

Instead of excluding Na^+^ and synthesising organic osmolytes, the ability of utilising Na^+^ can be considered to be a more effective trait in conferring salinity tolerance. Although accumulating excessive amount of Na^+^ can become toxic for plants, Na^+^ uptake is desirable because this element is highly soluble and easily available (especially under salinity) for plants to increase osmotic pressure, absorb water, and sustain turgor [27,28]. A sharp increase of xylem Na^+^ loading and shoot Na^+^ accumulation can be an efficient means of osmotic adjustment, and this Na^+^ utilisation mechanism has been reported from halophytes and salinity-tolerant barley genotypes [29,30,31,32]. Excessive Na^+^ elevation in the cytosol causes toxicity; therefore, effective Na^+^ sequestration into vacuoles mediated by tonoplast Na^+^(K^+^)/H^+^ exchanger (NHX) has to be accompanied with the above mechanisms of Na^+^ utilisation. Recently, a need for a shift from crop breeding for Na^+^ exclusion towards conferring superior traits benefitting from Na^+^ called “halophytism” was suggested [33]. 

The only halophytic relative of wild rice species, *O. coarctata,* is known to grow under high level of salinity (20–40 ds m^−1^) that is lethal for cultivated rice (*O. sativa*) species [34,35,36]. *O. coarctata* has long been known to maintain low leaf Na^+^/K^+^ ratio [37], showing greater Na^+^ accumulation in the root rather than the shoot under salinity [38]. Secretion of Na^+^ via external microhairs [39], efficient performance of NHX [40], and a high transport capacity of HKT1;5 [41] are considered to contribute to superior ionic homeostasis under salinity in this species. Due to high salinity tolerance within the genus of *Oryza*, *O. coarctata* has been considered as an important resource of gene pools to improve salinity tolerance in rice cultivars [35,41]. However, detailed mechanisms of maintaining Na^+^ homeostasis in this species have been less understood due to the limited number of studies at the cellular level. 

We hypothesise that *O. coarctata* possesses mechanisms, wherein Na^+^ is utilised rather than excluded, for adapting to a saline environment. To test this hypothesis, we compared a range of physiological variables (e.g., biomass change, relative water content, chlorophyll content, and stomatal conductance) between salt-grown cultivated (*O. sativa*) and wild (*O. coarctata*) rice species and linked them with kinetics of Na^+^ transport in plant roots; Na^+^ concentrations in root, leaf, and xylem sap; and expression of SOS1, NHX, and HKT1 transporter genes. The overall research aim was to explore the mechanisms of Na^+^ uptake, exclusion, and translocation differentiating Na^+^ homeostasis between these two rice species.

## 2. Results

### 2.1. Biomass Change, Relative Water Content, and Physiological Responses

**Figure 1:**
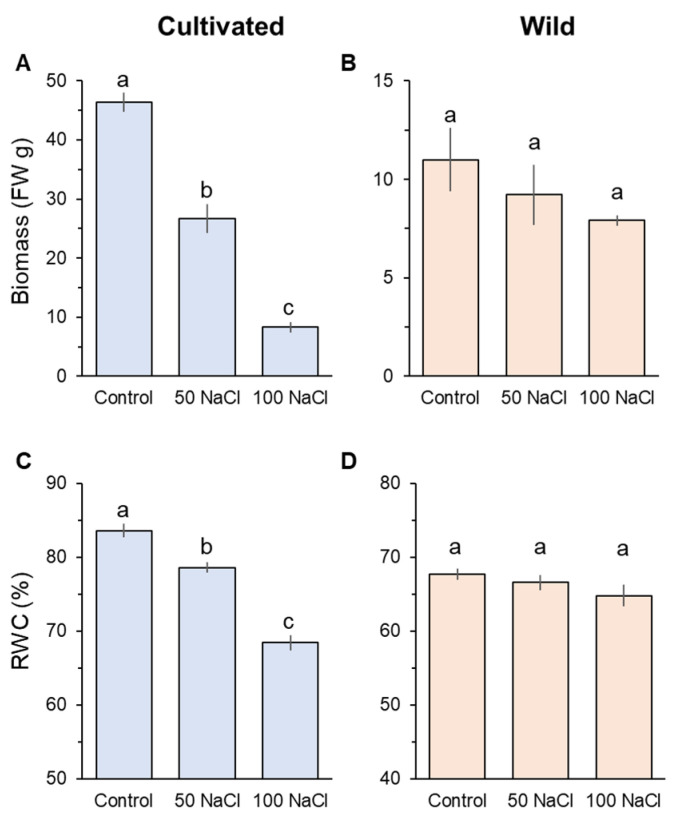
Whole-plant biomass change in fresh weight measured from cultivated (**A**) and wild (**B**) rice species. Shoot relative water content (RWC) of cultivated (**C**) and wild rice species (**D**). Seedlings were exposed to 0 (control), 50, or 100 mM NaCl for four weeks. Different letters indicate significant differences (*p* < 0.05, one-way ANOVA followed by LSD tests). Mean ± SE (*n* = 6).

After four weeks of salinity treatment, prominent differences were observed in plant biomass (FW) and relative water content (RWC) between cultivated and wild rice species (Figure 1). Cultivated rice significantly (*p* < 0.05) declined in biomass and RWC in response to the increase of salinity levels (Figure 1A,C). Notably, cultivated rice treated with 100 mM NaCl showed an eightfold decline in biomass compared with its controls (Figure 1C). In contrast, both 50 and 100 mM NaCl treatments did not significantly decrease both biomass and RWC compared with the control in wild rice (Figure 1B,D).

**Figure 2:**
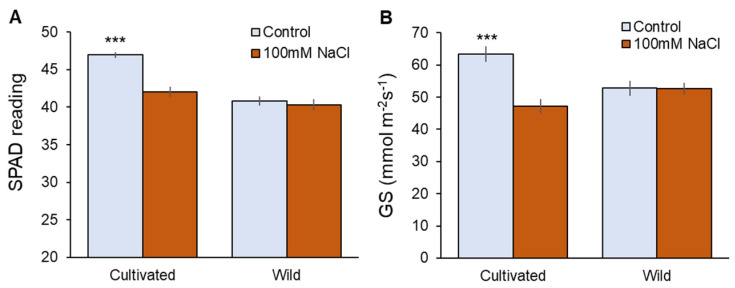
Physiological responses to two weeks of salinity (100 mM NaCl) treatment in cultivated and wild rice species. (**A**) SPAD value (chlorophyll content); (**B**) Gs (stomatal conductance). Asterisks indicate significant differences within the plant species (*** significant at *p* < 0.001, Student’s *t*-tests). Mean ± SE (*n* = 10).

Physiological characteristics were also affected only in cultivated rice in response to salinity (Figure 2). Two weeks of 100 mM NaCl treatment significantly (*p* < 0.001) reduced chlorophyll content (Figure 2A) and stomatal conductance (Figure 2B) in cultivated rice, while wild rice showed almost the same values between control and salt-treated plants (Figure 2A,B). The above observations suggest that wild rice is considerably more salinity-tolerant at the whole-plant level compared with cultivated rice.

### 2.2. Root, Leaf, and Xylem Sap Na^+^ Concentrations 

**Figure 3:**
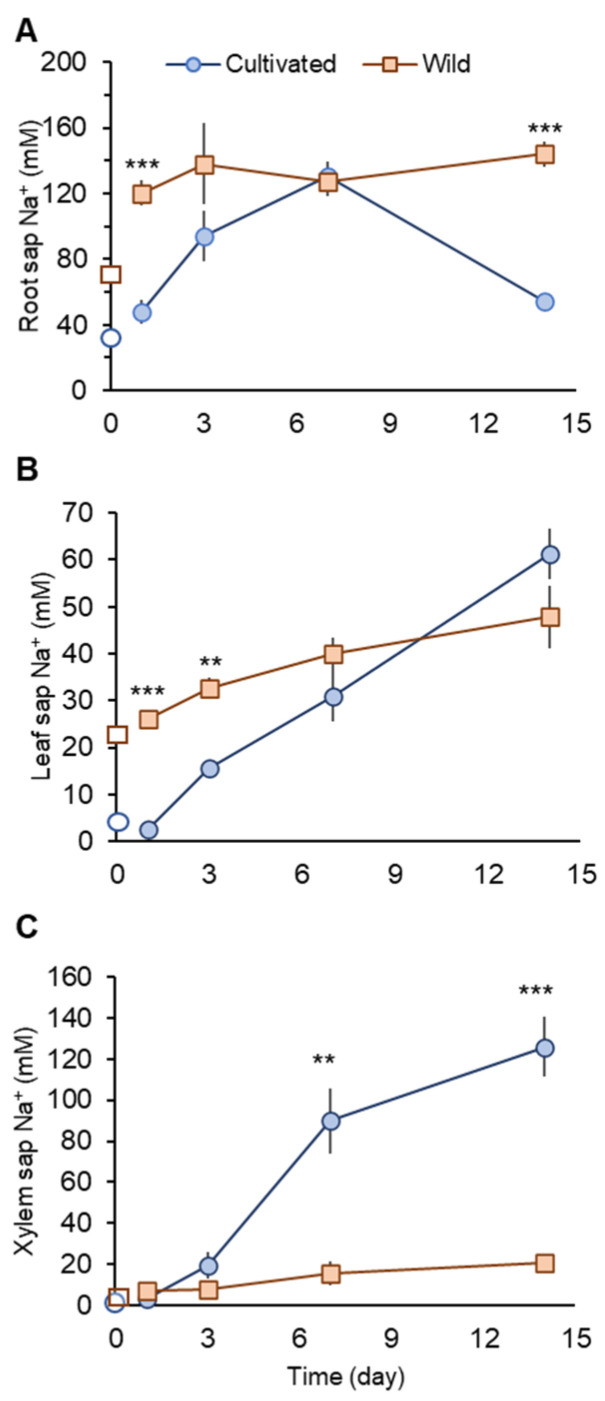
Na^+^ concentrations in root (**A**), leaf (**B**), and xylem sap (**C**) in cultivated and wild rice species under 100 mM NaCl treatments at different time points after the commencement of salinity. Open symbols describe Na^+^ concentrations before salinity onset. Asterisks indicate significant differences between plant species within the same harvest day. (** *p* < 0.01; *** *p* < 0.001, Student’s *t*-tests). Mean ± SE (*n* = 3–6).

Under non-saline conditions, wild rice showed about two- and fourfold higher Na^+^ concentrations in root and leaf sap, respectively, compared with cultivated rice (Figure 3A,B). 

One day after the commencement of salinity treatment (day 1), cultivated rice showed significantly (*p* < 0.001) smaller Na^+^ concentrations in root and leaf sap than those in wild rice (Figure 3A,B). At the same time (day 1), Na^+^ concentration in xylem sap in cultivated rice showed only a very marginal increase (not significant at *p <* 0.05), while in wild rice, this increase was substantial (threefold; from 2.55± 0.49 in control plant to 7.28 ± 1.19 mM in salt-treated plant; significant at *p* < 0.05). Thus, at the beginning of a salinity event, cultivated rice may have mechanisms operative to prevent root Na^+^ uptake and xylem Na^+^ loading. In contrast, wild rice showed increased xylem Na^+^ loading and Na^+^ transport to the shoot.

When salinity stress was prolonged, cultivated rice Na^+^ concentration in the root sap increased until day 7, but dropped sharply at day 14 to become significantly (*p* < 0.001) lower than that seen in wild rice (Figure 3A). This sharp drop of root sap Na^+^ in cultivated rice can be accounted for by increased Na^+^ transport to the shoot. Leaf sap Na^+^ concentration in cultivated rice progressively increased over the period of salinity treatment, with a sharp increase after day 7 (Figure 3B). Xylem sap Na^+^ concentration in cultivated rice was not significantly different compared with wild rice until day 3. However, a sharp and substantial increase in the xylem sap Na^+^ concentration in cultivated rice was observed at day 7, with values being significantly (fourfold, *p* < 0.01) higher than for wild rice (Figure 3C). The increase in xylem sap Na^+^ concentration in cultivated rice was observed until day 14 (Figure 3C). In contrast, although salt-treated wild rice showed approximately twofold higher root sap Na^+^ concentrations over the period of salinity treatment compared with control, the variation in root sap Na^+^ concentrations were not as large (Figure 3A). Therefore, wild rice may possess superior ability to retain Na^+^ in the root under prolonged salinity compared with cultivated rice. Further, wild rice also maintained rather stable Na^+^ concentrations in leaf and xylem sap over the period of salinity treatment (Figure 3B,C).

### 2.3. Transcriptional Analysis of Genes Related to Na^+^ Transport

**Figure 4:**
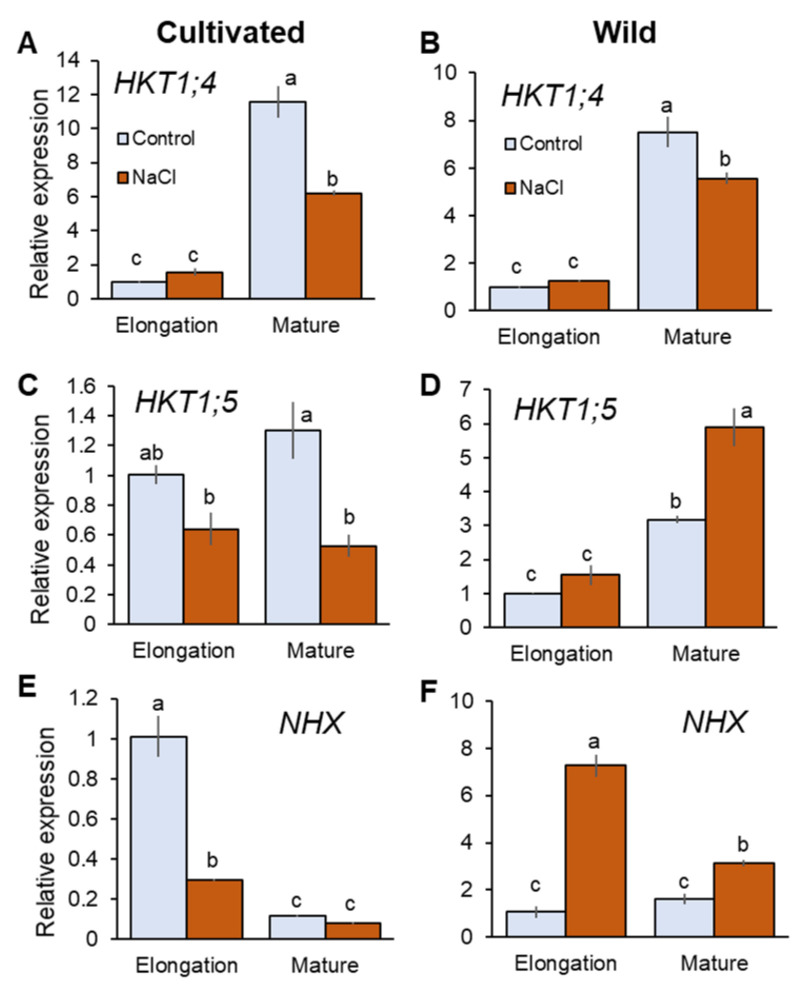
RT-qPCR analysis of the gene expressions of *HKT1;4* (**A**,**B**), *HKT1;5* (**C**,**D**), and *NHX1* (**E**,**F**) in mature and elongation root zones of cultivated and wild rice species under control and salinity (100 mM NaCl, 48 h) conditions. Different letters indicate significant differences (*p* < 0.05, one-way ANOVA followed by LSD tests). Mean ± SE (*n* = 3).

HKT1;4 and HKT1;5 are known to mediate retrieval back of Na^+^ from the xylem that contribute to reduce shoot Na^+^ accumulation in rice [6,42]. Salinity treatment did not significantly (*p* < 0.05) change expression of *HKT1*;4 in the elongation root zone (EZ), but significantly downregulated it in the mature zone (MZ) in both species (Figure 4A,B). It is reported that HKT1;4 mediates Na^+^ unloading in a range of conditions (submillimolar Na^+^ to high salinity) in cultivated rice [42]. Further, OsHKT1;4 has been suggested to have a more prominent role in mediating Na^+^ unloading in the leaf sheath at the reproductive stage, preventing over-accumulation of Na^+^ in the leaf blade under salinity [43]. Therefore, it may be considered that HKT1;4 has a very minor or no role in Na^+^ transport into xylem parenchyma cells under saline conditions tested here. *HKT1*;5 expression was downregulated in cultivated rice but upregulated in wild rice by salinity (Figure 4C,D). In MZ, cultivated rice showed 59.2% decrease in *HKT1*;5 expression in response to salinity (significant at *p* < 0.05), in contrast to wild rice that showed an 85.6% increase in *HKT1*;5 expression (significant at *p* < 0.05, Figure 4C,D). Tonoplast Na^+^/H^+^ antiporter (NHX1) mediates Na^+^ sequestration into vacuoles to reduce excessive increase of cytosolic Na^+^ concentration [44]. Having a dual affinity for both Na^+^ and K^+^ NHX1 also catalyses K^+^/H^+^ exchange at the tonoplast membrane [45]. *NHX1* expression was significantly increased in both root zones of wild rice (sevenfold and twofold in EZ and MZ, respectively; Figure 4F). In contrast, cultivated rice showed downregulated *NHX1* expression in response to salinity (Figure 4E)—for example, there was a threefold reduction in *NHX1* expression (significant at *p* < 0.05) in EZ under salinity in the cultivated rice. Thus, wild rice showed greater expressions of *HKT1;5* and *NHX* under salinity compared with cultivated rice.

### 2.4. Ion Flux Measurements on the Root Epidermis in Response to NaCl

#### 2.4.1. NaCl-Induced Na^+^ Influx

**Figure 5:**
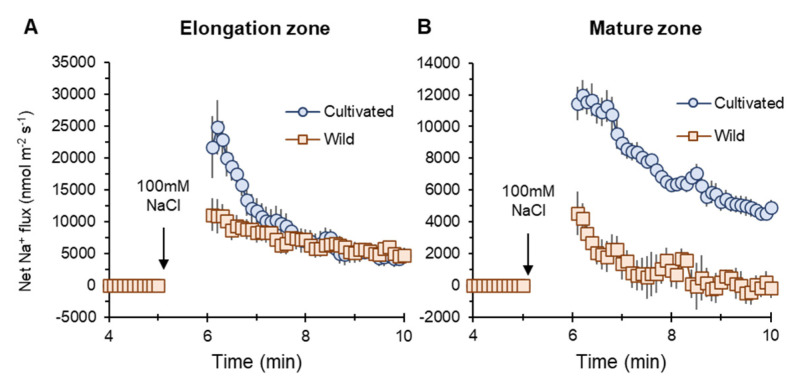
Transient net Na^+^ flux measured from elongation (**A**) and mature (**B**) root zones of cultivated and wild rice species in response to 100 mM NaCl application. Mean ± SE (*n* = 7–10).

**Figure 6:**
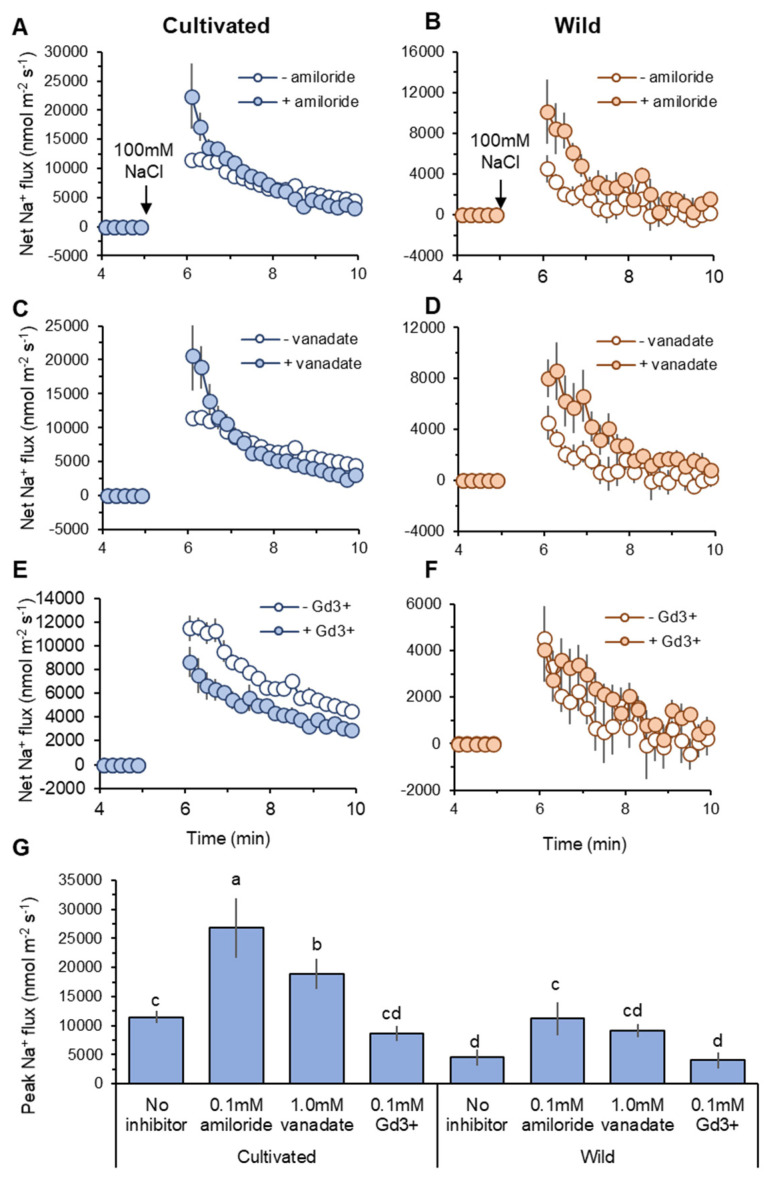
Pharmacological analysis of transient net Na^+^ flux measured from mature root zones of cultivated and wild rice species in response to 100 mM NaCl application. Roots were pre-treated for 1 h with one of the following known inhibitors: 0.1 mM amiloride, an inhibitor of Na^+^/H^+^ exchanger (**A**,**B**); 1 mM sodium orthovanadate (vanadate), H^+^-ATPase blocker (**C**,**D**); 0.1 mM GdCl_3_ (Gd^3+^), non-selective cation channel NSCC blocker (**E**,**F**). Peak Na^+^ flux identified as maximum flux value during measurements (**G**). Different letters indicate significant differences (*p* < 0.05, one-way ANOVA followed by LSD tests). Mean ± SE (*n* = 6–8).

Na^+^ influx into elongation (EZ) and mature root zones (MZ) of both two rice species were induced by salt (100 mM NaCl) application. However, cultivated rice showed higher influx than wild rice (Figure 5A,B). The peak value of net Na^+^ flux in MZ in the cultivated rice was much higher (about twofold, significant at *p* < 0.05) than that in wild rice (marked as “no inhibitor” in Figure 6G).

#### 2.4.2. SOS1 Operations in Reducing Net Na^+^ Influx

Cellular Na^+^ exclusion in plants is mediated by Na^+^/H^+^ exchanger (SOS1) fuelled by H^+^-ATPase activity at the root plasma membrane [12,22]. Thus, net Na^+^ influx into the root can be explained by the difference between unidirectional Na^+^ entry into the root and SOS1-mediated Na^+^ efflux from the root. Pharmacological experiments revealed that net Na^+^ efflux was decreased by both amiloride (an inhibitor of Na^+^/H^+^ exchanger: SOS1) and sodium orthovanadate (vanadate: H^+^-ATPase blocker) pre-treatments in both species (Figure 6A–D). The peak Na^+^ flux values were significantly (*p* < 0.05) increased by amiloride and vanadate pre-treatments compared with no-inhibitor within the same species, except vanadate pre-treatment in wild rice (Figure 6G). This suggests activity of SOS1 fuelled by H^+^-ATPase at the root plasma membrane in both species. Compared with wild rice, cultivated rice showed a greater shift towards net Na^+^ influx caused by SOS1 inhibition. The increases in peak Na^+^ flux caused by amiloride pre-treatment (relative to no-inhibitor) were 15,346 and 6685 nmol m^−2^ s^−1^ in cultivated and wild rice, respectively (Figure 6G). Likewise, cultivated rice also showed greater increase of peak Na^+^ influx by vanadate pre-treatment than wild rice (7414 vs. 4636 nmol m^−2^ s^−1^, Figure 6G). This suggests that cultivated rice relies more on SOS1 activity for cellular Na^+^ extrusion at the root epidermis under salinity than wild rice.

#### 2.4.3. Na^+^ Influx through NSCC

Non-selective cation channels (NSCC) have been known as a major pathway of Na^+^ entry into the root [46]. Although GdCl_3_ (Gd^3+^; NSCC blocker) pre-treatment did not significantly change peak Na^+^ influx values in two species, it reduced the peak of Na^+^ influx in cultivated rice by 24.6%, while in wild rice, this reduction was only 10.3% (Figure 6G). Moreover, kinetics of net Na^+^ influx was always smaller in the root treated with Gd^3+^ relative to “no-inhibitor” control in cultivated rice after salt application (Figure 6E), while Gd^3+^-induced difference in net Na^+^ flux in wild rice was less obvious (Figure 6F). These observations suggest that NSCCs may play a smaller role in Na^+^ uptake in wild rice compared with cultivated rice.

#### 2.4.4. H^+^ Flux in SOS1 Operations

**Figure 7:**
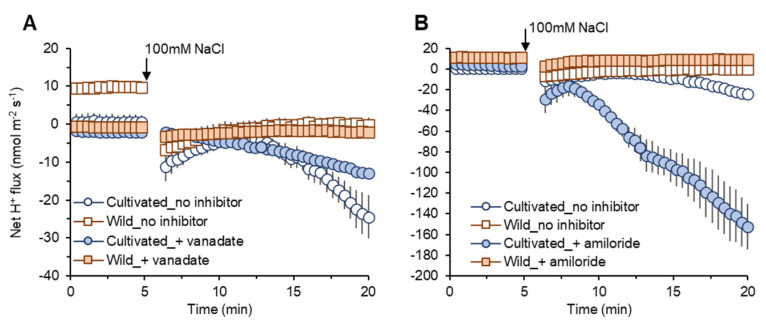
Transient net H^+^ flux measured from mature root zones of cultivated and wild rice species in response to 100 mM NaCl application with 1 h of pre-treatment of known inhibitors: 1 mM sodium orthovanadate (vanadate), H^+^-ATPase blocker (**A**); 0.1 mM amiloride, an inhibitor of Na^+^/H^+^ exchanger (**B**). Mean ± SE (*n* = 5–6).

Vanadate pre-treatment induced only slight reductions in net H^+^ efflux in both species (Figure 7A). However, amiloride pre-treatment induced a much more prominent increase in net H^+^ efflux (reduction in the amount of H^+^ exchanged by Na^+^ in SOS1 operations) in cultivated rice compared with wild rice (Figure 7B). These data can be taken as evidence for higher SOS1 activity in cultivated rice to reduce Na^+^ uptake compared with wild rice.

### 2.5. Analysis of SOS1 Functional Activity

To assess functional activity of SOS1 in the root plasma membrane, we used the so-called “recovery protocol” [47]. The idea behind it is that the root is exposed to salinity and allowed to accumulate Na^+^ for some time and induce expression of SOS1 genes required for its extrusion. The roots are then transferred to Na^+^-free media and, after transient processes in the apoplast (Donnan system) are over, the magnitude of net Na^+^ efflux reflects the functional activity of SOS1-like exchanger.

**Figure 8:**
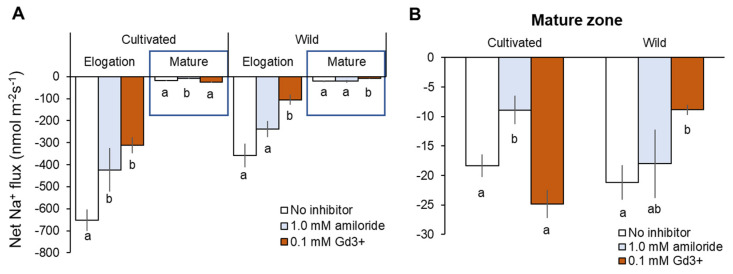
Na^+^ efflux from elongation and mature root zones (**A**) and only mature root zone (**B**) of cultivated and wild rice after the removal of 100 mM NaCl (48 h treatment) with known inhibitors: 0.1 mM amiloride, an inhibitor of Na^+^/H^+^ exchanger; 0.1 mM GdCl_3_ (Gd^3+^), NSCC blocker. Steady-state net Na^+^ flux was measured 20 min after NaCl removal. Different letters indicate significant differences within the same root zone in the same species (*p* < 0.05, one-way ANOVA followed by LSD tests). Mean ± SE (*n* = 5–6).

Consistent with reported expression of SOS1 genes, both rice species showed dramatically higher net Na^+^ efflux in the root elongation zone (EZ) than mature zone (MZ) (Figure 8A). In EZ, about 80% greater net Na^+^ efflux was observed from cultivated rice root without inhibitor than those in wild rice (cultivated rice: −651 ± 48 vs. wild rice: −359 ± 53 nmol m^−2^ s^−1^, Figure 8A). Amiloride (SOS1 inhibitor) pre-treatment significantly (*p* < 0.05) reduced net Na^+^ efflux in cultivated rice in both root zones, while amiloride-induced decrease of Na^+^ efflux in wild rice was not significant in both root zones (Figure 8A,B). Wild rice showed a significant decrease in net Na^+^ efflux by Gd^3+^ (NSCC blocker) pre-treatment in both root zones, while cultivated rice showed Gd^3+^-induced decrease of Na^+^ efflux (with significance, *p* < 0.05) in only EZ (Figure 8A,B). The above observations suggest that cultivated rice mediates greater Na^+^ efflux than wild rice, and therefore cultivated more relies on SOS1 activity for Na^+^ exclusion compared with wild rice. On the other hand, passive Na^+^ leakage through NSCC (rather than active Na^+^ exclusion by SOS1) largely contributed to Na^+^ efflux from the root of wild rice.

**Figure 9:**
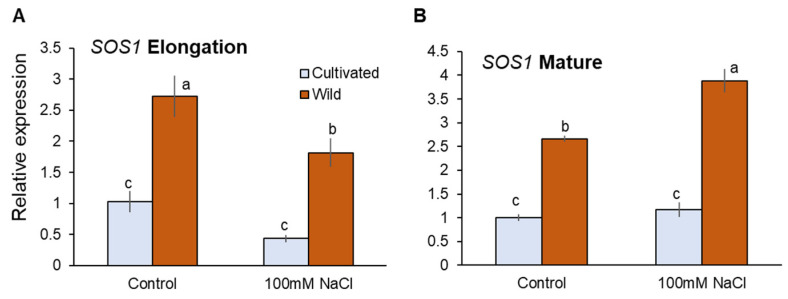
RT-qPCR analysis of the gene expressions of SOS1 in mature (**A**) and elongation (**B**) root zones of cultivated and wild rice species under control and salinity (100 mM NaCl, 48 h) conditions. Different letters indicate significant differences (*p* < 0.05, one-way ANOVA followed by LSD tests). Mean ± SE (*n* = 3).

Transcriptional analysis showed that *SOS1* expressions were significantly higher in wild rice than cultivated rice under both control and salinity (100 mM NaCl for 48 h) conditions (Figure 9A,B). Salinity-induced changes in *SOS1* expressions were not significant in both root zones of cultivated rice, while those in wild rice were significant downregulation and upregulation in EZ and MZ, respectively (Figure 9A,B). As the above differences in SOS1 transcriptions can hardly explain actual SOS1 operations observed from Na^+^ flux measurements, it appears that SOS1 activities might be regulated at the post-translational rather than transcriptional level.

## 3. Discussion

### 3.1. Leaf Tissue Na^+^ Tolerance Observed in Wild Rice Is Highly Important for the Overall Salinity Tolerance in this Species 

Wild rice possesses fourfold higher leaf sap Na^+^ concentration than cultivated rice under un-salinised (0 mM NaCl) conditions (Figure 3B). Halophytes typically possess a greater amount of Na^+^ in their leaf tissues compared with glycophytes [29], and this phenomenon is attributed to a likely role of Na^+^ in maintenance of cell turgor [28]. Two weeks of salinity (100 mM NaCl) treatment increased leaf sap Na^+^ concentration in both rice species, with no significant difference between them (Figure 3B). However, a significant reduction in chlorophyll content was observed in cultivated rice (Figure 2A) that is a typical symptom of Na^+^ toxicity [48]. This was not observed in wild rice (Figure 2B), indicating its higher tissue tolerance to Na^+^ [49] that may be conferred by a superior sequestration of Na^+^ into vacuoles [40,50]. Superior tissue tolerance has been shown to confer salinity tolerance in the wild rice species *O. rufipogon* [50], and this trait has been suggested to be targeted for rice breeding instead of Na^+^ exclusion [49,50]. Here, leaf tissue Na^+^ tolerance was reported as being a hallmark for one of the most salt tolerant rice species, *O. coarctata*, validating the above conclusion. 

### 3.2. Different Means of Osmotic Adjustment Differentiated Stress Tolerance between Two Species

Onset of salinity treatment also triggers osmotic stress, causing plant dehydration. Plants increase osmotic pressure in the cells and regain turgor in response to osmotic stress in a process called osmotic adjustment. There are two means of osmotic adjustment, namely, synthesis of organic osmolytes (compatible solutes) and accumulation of inorganic ions within cells [30,51]. At the early stage of salinity (one day after the stress onset), a significant (*p* < 0.05) increase in xylem sap Na^+^ concentration was observed in wild rice, but not in cultivated rice. Thus, it is reasonable to suggest that wild rice relies on Na^+^ transfer to the shoot for osmotic adjustment, while cultivated rice heavily relies on de novo synthesis of organic osmolytes and tries to minimise xylem Na^+^ loading.

As biosynthesis of organic osmolytes is a highly energy-consuming process, it leads to growth penalties under prolonged salinity [20,52]. In addition to osmotic adjustment, stomatal operation is also a critical factor under osmotic stress conditions. In response to drought or salinity stress, stomatal closure is induced by ABA accumulation to conserve water in plants [53], and only cultivated rice significantly (*p* < 0.001) reduced stomatal conductance under salinity (Figure 2B). Reduced stomatal conductance results in a decrease of the ability of a plant to assimilate CO_2_, thus limiting photosynthesis and plant growth [50,54,55]. Despite energy cost by de novo synthesis of organic osmolytes and reduced CO_2_ assimilation due to stomatal closure, cultivated rice showed symptoms of dehydration (significant decrease of RWC at *p* < 0.05, Figure 1C). Osmotic adjustment by means of Na^+^ accumulation is quick, energy-saving, and more efficient compared with de novo synthesis of organic osmolytes [20,30,56] and may be the reason that wild rice did not show a significant dehydration (Figure 1D) and stomatal closure (Figure 2B). Thus, utilisation of Na^+^ for osmotic adjustment is a significant trait differentiating tolerance to salinity induced osmotic stress between rice species.

### 3.3. Maintenance of Na^+^ Homeostasis under Long-Term Salinity Is the Key Determinant of Overall Salinity Tolerance in Wild Rice

Na^+^ toxicity is considered as a main constraint imposed by the long-term salinity stress [57]. In addition to the response to salinity induced osmotic stress (explained in the above section), the two rice species differently controlled Na^+^ uptake and transport to avoid Na^+^ toxicity. 

Cultivated rice showed significantly (*p* < 0.001) lower Na^+^ concentrations in the root sap than wild rice at the beginning (day 1) of the salinity treatment (Figure 3A) that may be explained by greater activities of Na^+^/H^+^ exchanger (SOS1) located at the root plasma membrane for mediating Na^+^ exclusion in this species (Figure 6 and Figure 8; see also the next section for more discussion). Cellular Na^+^ exclusion mediated by SOS1 activity fuelled by H^+^-ATPase is an energy-consuming process as well as de novo synthesis of organic osmolytes for osmotic adjustment [19]. This suggests that cultivated rice expends a substantial amount of energy, leading to depletion of ATP pool when salt stress is prolonged. At a later stage of salinity imposition, cultivated rice may be low on available energy and therefore unable to control significant Na^+^ entry into the root and thus Na^+^ transport to the shoot, resulting in severe Na^+^ toxicity leading to significant biomass reductions (Figure 1A). The above pattern of Na^+^ transport under salinity observed in cultivated rice is also typically observed in salinity sensitive glycophytic species [29,30]. 

Compared with cultivated rice, wild rice has more efficiently control over Na^+^ transport during the imposition of salinity. Once osmotic adjustment by means of Na^+^ accumulation is achieved, wild rice maintains rather stable leaf and xylem sap Na^+^ concentrations (from day 3; Figure 3B,C), and this is coupled with root Na^+^ accumulation that is significantly greater than in cultivated rice, two weeks after the onset of salinity stress (Figure 3A). This pattern of Na^+^ transport is effective to avoid shoot Na^+^ toxicity. Although a functional role of OsHKT1;5 is still questioned due to direct measurements of Na^+^ flux from root stele [58], OsHKT1;5 within the *SKC1* locus is suggested to mediate xylem Na^+^ unloading that reduces shoot Na^+^ accumulation under salinity [59]. In addition, effective Na^+^ sequestration into root vacuoles through NHX was found to be a key determinant of salinity tolerance in barley and wheat [21,60]. As only wild rice shows significantly upregulated *HKT1*;5 and *NHX1* expressions in the root in response to salinity (Figure 4C–F), it is plausible to suggest that this species may transfer an excessive amount of Na^+^ from the xylem to root vacuoles. For Na^+^ in root vacuoles to be retained for avoiding Na^+^ toxicity, effective control of Na^+^ back-leak into cytosol [61] is required and is suggested to operate in wild rice. The above mechanisms of root Na^+^ sequestration rather than exclusion observed in wild rice may be a critical determinant of salinity tolerance, allowing this species to maintain normal metabolism and plant growth under long-term salinity (Figure 1B).

### 3.4. Smaller Net Na^+^ Entry in Wild Rice Root Is Not Attributable to SOS1 Activity

In response to salt (100 mM NaCl) application, net Na^+^ influx into the roots of both rice species was observed in electrophysiological experiments (Figure 5). Na^+^ enters into the root through major two pathways, namely, NSCC and HKT [46]. Sensing Na^+^ entry results in the elevation of cytosolic Ca^2+^, cGMP, and H_2_O_2_ production [62,63] that activates H^+^-ATPase-mediating H^+^ efflux fuelling SOS1 activity to exclude Na^+^ from the cytosol [64,65]. Due to the above Na^+^ efflux system, the difference between Na^+^ entry and Na^+^ exclusion can explain the observed net Na^+^ influx into the roots of both rice species. In response to transient application of salt stress, wild rice showed much lesser net Na^+^ influx compared with cultivated rice (Figure 5A,B). However, this smaller net Na^+^ influx was not due to greater activity of SOS1-mediated Na^+^ extrusion. Both amiloride (a blocker of SOS1 exchanger) and vanadate (H^+^-ATPase inhibitor) pre-treatments suggested greater involvement of SOS1 activity in cultivated rice than in wild rice (Figure 6A–D,G and Figure 7A,B). Na^+^ influx into root cells under salinity is a passive process [20], as both low cytosolic Na^+^ concentrations and the negative electrical difference at the plasma membrane readily mediate Na^+^ movement into cells [66]. Under non-saline conditions, wild rice possesses much greater root sap Na^+^ concentration (Figure 3A) and less negative membrane potential (data are not shown) compared with cultivated rice. Therefore, wild rice may be able to allow smaller degree of salinity-induced Na^+^ gradient moving into the plasma membrane of root cells than cultivated rice (Figure 6E,F), thus showing smaller net Na^+^ influx in response to a sudden increase of external salt concentration.

### 3.5. Limiting Na^+^ Exclusion by SOS1 Activity under Long-Term Salinity Is Crucial to Improve Salinity Tolerance in Rice Species

Na^+^ efflux measurements in Na^+^-free solution after the removal of salt (the so called “recovery protocol”; [22,47] revealed the difference of root Na^+^ efflux mechanisms under long-term salinity (100 mM NaCl for 48 h) between cultivated and wild rice. A considerable degree of Na^+^ efflux from cultivated rice root was mediated by amiloride-sensitive SOS1 activity, while measured Na^+^ efflux from wild rice root was mostly due to passive Na^+^ movement through Gd^3+^-sensitive NSCC (Figure 8A,B). Moreover, in the root elongation zone (where SOS1 is predominantly located; [60,67], significantly larger Na^+^ efflux was observed from cultivated rice than wild rice. Therefore, greater activity of SOS1 for Na^+^ extrusion in cultivated rice under salinity was clearly observed from electrophysiological experiments. However, transcriptional changes in *SOS1* did not correlate with the observations at a functional level (Figure 9A,B). This is consistent with previous observations that the actual operation of SOS1 protein activity at a functional level does not always correlate with changes in transcript levels [22]. Further posttranslational mechanisms have been shown to be a major control point determining SOS1 activity in plants [68].

As mentioned in the previous sections, the issue with cellular Na^+^ exclusion mediated by SOS1 activity fuelled by sharp H^+^ gradient comes with a high energy cost. For this transporter to operate efficiently, for each Na^+^ ion expelled across the plasma membrane, one H^+^ ion needs to be extruded via H^+^-ATPase (every H^+^ extrusion hydrolyses one ATP; [19,20]). Therefore, a considerable energy penalty is imposed on cultivated rice due to the existence of the above-mentioned “futile cycle” at the root plasma membrane. Moreover, a passive apoplastic pathway of Na^+^ entry into the root named bypass flow in *O. sativa* species [7,23,24,25,26] may impose further detrimental effects, due to a futile cycle. It is therefore plausible to suggest that wild rice may possesses a superior ability to sequester Na^+^ into root vacuoles and limit passive Na^+^ entry through Na^+^-permeable channels/transporters under salinity, thus making SOS1-mediated Na^+^ extrusion playing only a small role in its overall salinity stress tolerance.

## 4. Materials and Methods

### 4.1. Plant Materials and Growth Conditions

Seeds of cultivated rice (*O. sativa* cv. Koshihikari) were obtained from Western Sydney University and then multiplied using glasshouse facilities at Tasmanian Institute of Agriculture, University of Tasmania, Hobart, Australia. Seeds were pre-germinated in an incubator (30 °C for three days) and sown into plastic cell trays filled with the standard potting mix containing 70% perlite and 30% sand using half strength Hoagland solution (see [69] for details). Two weeks after the sowing of pre-germinated seeds, young seedlings (three to four leaves stage) of cultivated rice were transplanted into the pots with the mixture of soil collected from University Farm, University of Tasmania, Cambridge, Tasmania, Australia (Chromosol, see [70] for details) and potting mix (65/35% *w*/*w*). The pot volume was 1.5 L, and each pot contained one plant. Wild rice (*O. coarctata*) seedlings were obtained from the Swaminathan Research Foundation (Chennai, India) and were propagated vegetatively. For vegetative propagation, *O. coarctata* seedlings were grown in 15 L plastic tubs with the mixture of soil and potting mix (see details above), which were filled with tap water up to the soil surface. Newly developed three to four leaf stages of wild rice seedlings (about one month after the emergence of the new plantlets) were carefully separated and transplanted to the pots under the same condition as for cultivated rice. Pots with transplanted cultivated or wild rice seedlings were placed in a 15 L plastic tub filled with tap water up to the soil surface (4× pots per tub). One week after transplanting, salinity stress was imposed for four weeks (0, 50, or 100 mM NaCl) by the replacement of tap water in the tubs by appropriate NaCl solutions. Plants were grown in the greenhouse (temperature: 25 ± 2 °C; 12 h light/12 h dark photoperiod).

### 4.2. Biomass Measurement and Relative Water Content

Whole-plant biomass (fresh weight: FW) was measured before transplanting to the pots, and all transplanted seedlings were labelled to be identified. After four weeks of salinity treatments (when specific effects of Na^+^ toxicity dominate), whole-plant FW was measured from labelled seedlings again, and the changes of FW (a biomass gain or loss) over the exposure to salinity were calculated. For measuring FW, seedlings were carefully removed from the growing medium, and their roots were then gently washed with a tap water to remove soil and quickly blotted. Roots and shoots were separated and weighted. Plant tissues were oven-dried, and shoot relative water content (RWC) was calculated.

### 4.3. Physiological Responses (Chlorophyll Content and Stomatal Conductance)

Chlorophyll content and stomatal conductance were measured from the second youngest fully expanded leaves two weeks after the commencement of salinity using SPAD-502 m (Konica Minolta, Osaka, Japan) and Decagon Leaf Porometer (Decagon Devices Inc., Pullman, WA, USA), respectively, as described in [71]. 

### 4.4. Root, Shoot, and Xylem Sap Na^+^ Analysis 

Root, leaf, and xylem saps were collected at different time points (1, 3, 7, and 14 days) after the commencement of salinity. Leaf sap was taken from the second youngest fully expanded leaves. Harvested roots were washed with 10 mM CaCl_2_ to remove apoplastic Na^+^ and quickly blotted. Harvested leaf and root samples were put into Eppendorf tubes and stored in the −20 °C freezer. Frozen samples were then thawed under the room temperature, and sap was obtained by hand-squeezing, as described in [71]. Xylem sap was collected using Scholander pressure chamber (Plant Moisture Systems, Santa Barbara, CA, USA). Each sample of xylem sap was collected from one to three plants per pot. Collected samples were weighed with 0.1 mg accuracy, then diluted and kept in the fridge. The content of Na^+^ and K^+^ in all sap samples was then measured using a flame photometry (model: PFP7, Jenway, Felsted, Dunmow, Essex, United Kingdom). 

### 4.5. RNA Isolation and Real-Time Quantitative PCR Analysis 

Excised root segments (3–5 cm long) were taken from the seedlings one month after transplanting of young seedlings into the pots (see the details of growing condition in Section 4.1). Seedlings were treated with 100 mM NaCl for 48 h before root harvest. Root segments were gently washed and blotted, and then cut into elongation and mature zone segments (1.0–2.0 and 12–15 mm from the root tip, respectively). Total RNA was isolated using RNAiso Plus (Takara, Shiga, Japan) as per the manufacturer’s protocol. First-strand cDNA synthesis was performed in a 20 µL reaction volume with 1 µg of total RNA and Superscript III (Invitrogen, Carlsbad CA, USA) at 42 °C for 60 min, followed by heat inactivation at 70 °C for 10 min. Real-time PCR (Quant Studio 6; Thermo Fisher, Waltham, MA, USA) was carried out in a reaction volume of 10 µL (1 µL of cDNA, 5 µL of Takara TB GreenTM Primix Ex TaqTM II (2×), 0.5 µL each of a given primer pair (final concentration of 250 nM each)) under the following cycling conditions: 95 °C (30 s), 40 cycles of denaturation at 95 °C (5 s), annealing and extension at 60 °C (30 s) in a 96-well optical reaction plate (Thermo Fisher, Waltham, MA, USA). The primer pairs listed in Appendix A were used to amplify fragments of indicated sizes for each target gene. Each real-time PCR reaction was performed in triplicate. Amplicon specificity was verified by melt curve analysis (60–95 °C at 40 cycles) and subsequent agarose gel electrophoresis. Gene expression was quantified using the comparative CT (2^−ΔΔCT^) quantitation method. Three biological replicates were used in all cases.

### 4.6. Ion Flux Measurements 

For electrophysiological experiments, newly developed underground adventitious roots from rhizomes of wild rice or crown of cultivated rice (crown root) were cut and taken one month after transplanting of young seedlings (three to four leaves stage) to the pots (see the details of growing condition in Section 4.1). Seedlings were grown in tap water until root harvest. Net ion fluxes were measured by using non-invasive ion-selective microelectrode (MIFE) technique (University of Tasmania, Hobart, Australia). Complete description of the theory of MIFE measurements, fabrication of ion-selective microelectrodes, and calibration processes have been previously explained in our past studies [72,73]. For preparation of H^+^-selective microelectrodes, commercially available ionophore cocktail (Merck, Germany; catalogue number 95291) was front-filled on the tips of electrodes. For Na^+^ measurement, an improved calixarene-based Na^+^ ionophore cocktail [74,75] was used.

Excised root segments (3–5 cm long) were carefully washed in a basic salt medium (BSM; 0.5 mM KCl, 0.1 mM CaCl_2_; pH 5.7, unbuffered) solution and then immobilised in the measuring chamber containing BSM. Fluxes of Na^+^ and H^+^ were measured from epidermal cells of elongation and mature root zones (1.0–2.0 and 12–15 mm from the root tip, respectively). Steady net ion fluxes were measured for five minutes in BSM solution, then salt treatment was applied to bring the final NaCl concentration (100 mM NaCl) in the measuring chamber. The resulting transient ion flux was recorded for up to 25 min. For the pharmacological experiment, excised root segments were pre-treated with known inhibitors (Appendix B) for one hour before ion flux measurement. Measurements were conducted from at least five individual plants

### 4.7. Measuring Na^+^/H^+^ Exchanger Activity 

To quantify activity of the plasma membrane Na^+^/H^+^ exchangers mediating Na^+^ extrusion from plant roots, we used a so called “recovery protocol” as described in the previous study [47]. Root segments (3–5 cm long) were cut and taken from the seedlings (see the details of growing condition and root harvest in Section 4.1 and Section 4.6) treated with 0 or 100 mM NaCl for 48 h before root harvest. Excised root segments were thoroughly and quickly washed with 10 mM CaCl_2_ to remove apoplastic NaCl and rinsed with double-distilled water. The roots were then transferred into Na^+^-free BSM solution and kept for 20 min, and net Na^+^ flux was measured for 3–5 min.

## Data Availability

Not applicable.

## Appendix A. List of Primers for RT-qPCR Analysis

**Oligos Name**	**Oligos (5′ → 3′)**
*OsHKT1; 4*	GACAGATCAATCCAGACCATCTC
	AGCCTcCCAAAGAACATCAc
*OcNHX1*	GAGAGGAGCTGTGTCGATTGc
	GGTAGCAGCAGCCTGATCAATG
*OcHKT1; 5*	ATTCTGGcTCCAACTGCTGIACT
	GTGAAGATCAGGTCCAAGTCCAT
*OcSOS1*	AGAAGTTCAAGAGGAATCCACCAT
	GGATCGTGCcATGTCCTTT

## Appendix B. List of Inhibitors for Pharmacological Experiments

**Name**	**Mode of Action**	**Concentration**
Amiloride	Na^+^/H^+^ exchanger inhibitor	0.l mM
Sodium orthovanadate (vanadate)	H^+^ - A1Pase blocker	l mM
GdC1_3_ (Gd^3+^)	NSCC blocker	0.1 mM
